# Evolution of xyloglucan-related genes in green plants

**DOI:** 10.1186/1471-2148-10-341

**Published:** 2010-11-05

**Authors:** Luiz Eduardo V Del Bem, Michel GA Vincentz

**Affiliations:** 1 Centro de Biologia Molecular e Engenharia Genética, Universidade Estadual de Campinas (UNICAMP), CP 6010, CEP 13083-875, Campinas, SP, Brazil; 2 Departamento de Biologia Vegetal, Instituto de Biologia, Universidade Estadual de Campinas (UNICAMP), CP 6109, CEP 13081-970, Campinas, SP, Brazil

## Abstract

**Background:**

The cell shape and morphology of plant tissues are intimately related to structural modifications in the primary cell wall that are associated with key processes in the regulation of cell growth and differentiation. The primary cell wall is composed mainly of cellulose immersed in a matrix of hemicellulose, pectin, lignin and some structural proteins. Xyloglucan is a hemicellulose polysaccharide present in the cell walls of all land plants (Embryophyta) and is the main hemicellulose in non-graminaceous angiosperms.

**Results:**

In this work, we used a comparative genomic approach to obtain new insights into the evolution of the xyloglucan-related enzymatic machinery in green plants. Detailed phylogenetic analyses were done for enzymes involved in xyloglucan synthesis (xyloglucan transglycosylase/hydrolase, α-xylosidase, β-galactosidase, β-glucosidase and α-fucosidase) and mobilization/degradation (β-(1→4)-glucan synthase, α-fucosyltransferases, β-galactosyltransferases and α-xylosyl transferase) based on 12 fully sequenced genomes and expressed sequence tags from 29 species of green plants. Evidence from Chlorophyta and Streptophyta green algae indicated that part of the Embryophyta xyloglucan-related machinery evolved in an aquatic environment, before land colonization. Streptophyte algae have at least three enzymes of the xyloglucan machinery: xyloglucan transglycosylase/hydrolase, β-(1→4)-glucan synthase from the celullose synthase-like C family and α-xylosidase that is also present in chlorophytes. Interestingly, gymnosperm sequences orthologs to xyloglucan transglycosylase/hydrolases with exclusively hydrolytic activity were also detected, suggesting that such activity must have emerged within the last common ancestor of spermatophytes. There was a positive correlation between the numbers of founder genes within each gene family and the complexity of the plant cell wall.

**Conclusions:**

Our data support the idea that a primordial xyloglucan-like polymer emerged in streptophyte algae as a pre-adaptation that allowed plants to subsequently colonize terrestrial habitats. Our results also provide additional evidence that charophycean algae and land plants are sister groups.

## Background

The cell shape and morphology of all plant tissues are a consequence of cell division and expansion throughout the plant's life cycle. Structural modifications in the primary cell wall (PCW) are key processes in the regulation of cell growth and differentiation. The PCW is a complex dynamic structure that shows spatial and temporal variability in composition and organization. Cell shape, size, and cell-cell adhesion are processes that rely on the coordinated action of enzymes involved in the synthesis, deposition, reorganization and selective disassembly of cell wall components. The ability to selectively modify the wall architecture is a major part of many processes such as cell growth, organ abscission, vascular differentiation, fruit softening and the response to pathogens [1-3].

The PCW consists primarily of cellulose immersed in a matrix of hemicellulose, pectin, lignin and some structural proteins [4,5]. Xyloglucan (XyG) is a well-characterized hemicellulose polysaccharide present in the cell walls of all spermatophytes [6]. Xyloglucan has also recently been found in the cell walls of non-vascular and seedless vascular plants [7]. XyG can also be stored as a reserve in cotyledons of many eudicots, such as nasturtium [8,9], *Tamarindus indica *[10], *Copaifera langsdorffii *[11] and *Hymenaea courbaril *[12].

**Figure 1:**
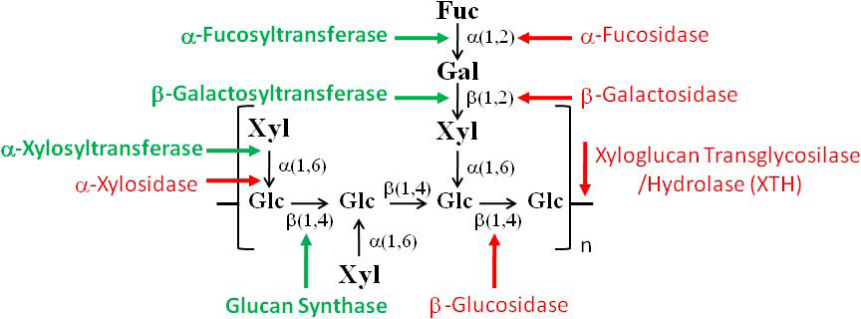
**Schematic representation of Xyloglucan (XyG) structure and related enzymatic activities**. An oligosaccharide of XyG (XXFG) is represented. β-(1-4)-glucan synthase produce the glucan backbone, α-Xylosyl Tranferase (XXT) acts on transfer xylose residues to the main backbone, β-Galactosyl Tranferase transfers galactose residue to xylose and α-Fucosyl Transferase transfer fucose residue to galactose. Xyloglucan Transglycosylase/Hydrolase (XTH) acts on hydrolysis of XyG oligosaccharides and/or XyG transglycosylation. α-Xylosidase removes the xylose residues, β-Galactosidase removes the galactose, α-fucosidase removes the fucose and β-Glucosidase mobilizes glucose monosaccharide from the main glucan backbone.

Xyloglucans have a main β-D-(1→4)-glucan backbone (denoted as G) generally branched with α(1→6)-linked D-xylopyranosyl (denoted as X) or β-D-galactopyranosyl (1→2)-D-xylopyranosyl residues (denoted as L). The presence of terminal fucosyl α-L-(1→2) units linked to branching β-D-galactosyl residues (denoted as F; for an example see Figure 1 and for nomenclature of XyG oligosaccharides see [13]) is the main difference between seed reserve XyG and structural XyG from the PCW of eudicot tissues [14].

Two main substitution patterns (XXXG and XXGG) occur in storage and structural eudicot XyG, although oligosaccharides containing five or six repeats (XXXXG and XXXXXG) have also been found in the XyG of seeds from the tropical tree *Hymenaea courbaril *([15,16]). The archetypical seed XyG of *Tamarindus indica *consists of XXXG, XXLG, XLXG and XLLG in a molar ratio of 1.4:3:1:5.4, respectively. However, these polysaccharides are not identical among plant groups. For example, the moss *Physcomitrella patens *and the liverwort *Marchantia polymorpha *synthesize XXGGG- and XXGG-type XyGs, respectively, with side chains that contain a β-D-galactosyluronic acid and a branched xylosyl residue. In contrast, hornworts synthesize XXXG-type XyGs that are structurally homologous to the XyGs synthesized by many seed-bearing and seedless vascular plants [7].

XyG is degraded *in vivo *by five hydrolases: β-galactosidase, α-xylosidase, β-glucosidase, xyloglucan transglycosylase/hydrolase (XTH) and α-fucosidase (Figure 1; [17,18]). Although the machinery involved in XyG degradation is relatively well-characterized [17], important details of the biosynthesis of this hemicellulose remain poorly understood. A number of enzymes participate in XyG biosynthesis, including β-(1→4)-glucan synthase, α-fucosyltransferases, β-galactosyltransferases and α-xylosyltransferases (Figure 1; [19]). Recently, only two genes for α-xylosyltransferases (*XXT1 *and *XXT2*) were found to be essential for the biosynthesis of XyG in *Arabidopsis *[20]. The double-mutant *xxt1*/*xxt2 *lacks detectable XyG and has aberrant root hairs, but is viable and has almost normal development. This finding challenges conventional models for the functional organization of PCW components [20].

An evolutionary analysis of the XyG-related machinery could provide new insights into the origin of this polymer during plant evolution, as well as information on the context in which it occurred. This knowledge could help to explain the role of XyG in plant adaptive features. In this work, we describe a comprehensive evolutionary analysis of the multigenic families of glycosyl hydrolases (β-galactosidase, α-xylosidase, β-glucosidase, XTH and α-fucosidase) and transferases (β-(1→4)-glucan synthase, α-fucosyltransferases, β-galactosyltransferases and XXT) involved in the biosynthesis, modification and degradation of XyG (Figure 1). Our results indicate that the XyG machinery is present in all embryophytic genomes and possibly emerged from the last common ancestor of the streptophytes (Charophyta algae + embryophytes). This inference suggests that the essential enzymes involved in XyG biosynthesis and turnover originated before land colonization by plants. This conclusion indicates that XyG is more than just a structural and mechanical molecule. Our data also provide additional evidence that streptophyte algae and land plants (Embryophyta) are sister groups.

## Results and Discussion

### Identification and phylogenetic analysis of XyG-related genes in green plant genomes

In order to identify genes related to XyG synthesis (β-(1→4)-glucan synthase, α-fucosyltransferases, β-galactosyltransferases and α-xylosyltransferases) and mobilization/modification (β-galactosidase, α-xylosidase, β-glucosidase, XTH, α-fucosidase; Figure 1) in green plants, we used previously characterized protein sequences as queries to perform blast searches using a self-employed algorithm (Additional File 1; see Methods for a complete list of the protein sequences used as queries). We also generated a sequence database containing the complete predicted proteomes and transcriptomes for 12 species (Viridiplantae 1.0 containing 365,187 protein sequences and Viridiplantae_nt 1.0 containing 403,380 EST sequences, respectively), including angiosperms (eudicots and monocots), seedless tracheophytes (Lycophyta), non-vascular plants (Bryophyta), and green algae (Chlorophyta). In addition, searches were also run against an EST database (ViridiESTs 1.0 containing 402,770 assembled EST sequences) that included sequences belonging to 29 species from taxonomic groups lacking complete genome information, such as basal and non-eudicot/monocot angiosperms, gymnosperms (Pinophyta, Cycadophyta, Ginkgophyta and Gnetophyta), seedless Tracheophyta (Pterydophyta), non-vascular plants (Marchantiophyta) and Streptophyta algae.

Using this strategy, we identified 862 XyG-related genes that included 293 XTH sequences, 133 β-galactosidases, 53 β-glucosidases, 24 α-xylosidases, 91 β-(1→4)-glucan synthases, 79 α-fucosyltransferases, 108 β-galactosyltransferases and 45 XXTs. We found two evolutionarily unrelated clusters of XyG-related α-fucosidase, one containing 22 sequences homologous to *Arabidopsis ATFXG1 *(At1g67830 - TAIR; [18]) and the other containing 14 sequences homologous to *Lilium longiflorum EBM II *(BAF85832 - GenBank; [21]). ESTs with less than 40% of the protein-based query coverage were excluded.

The relationships between genes can be represented as a system of homologous families that include orthologs and paralogs [22]. Orthologs are genes in different species that evolved from a common ancestral gene through speciation whereas paralogs are genes sharing a common ancestral gene that duplicated within the genome [23]. Orthologs normally retain their original function during evolution whereas paralogs can evolve new functions that may or may not be related to the original one. Consequently, the identification of orthologs is critical for the reliable prediction of gene functions in newly sequenced genomes. This identification is equally important for phylogenetic analysis because interpretable phylogenetic trees can generally be constructed only within sets of orthologs [23,24]. A complete list of orthologs is also a prerequisite for meaningful comparisons of genome organization [22].

The **po**ssible **g**roups of **o**rthologs (PoGOs) were established by using two phylogenetic analyses that involved the amino acid sequences. The first analysis was based on the p-distance (number of differences/number of aligned residues) and PAM 001 matrix [25] and used the neighbor-joining tree building method (NJ; [26]) while the second analysis was based on the maximum likelihood (ML; [27]). We also sought for shared derived ancestral intron positions in *Arabidopsis *(eudicot), sorghum (monocot), *Selaginella *(Lycophyte) and *Physcomitrella *(Bryophyte) since this information was useful in inferring the evolutionary relationships between homologous groups. The results for intron positions generally agreed with the phylogenetic analysis (data not shown). This combination of analyses yielded a comprehensive evolutionary profile of the enzymes involved in XyG synthesis and turnover. In the following sections, we present evidence that the complete set of enzymes involved in XyG synthesis and mobilization is present among all embryophytic lineages and that some of them emerged within streptophytes (XTH and β-(1→4)-glucan synthase) and chlorophyte algae (α-xylosidase).

### XTH originated in the last common ancestor of streptophytes, before land colonization, and was amplified through several lineage-specific events in embryophytes

**Figure 2:**
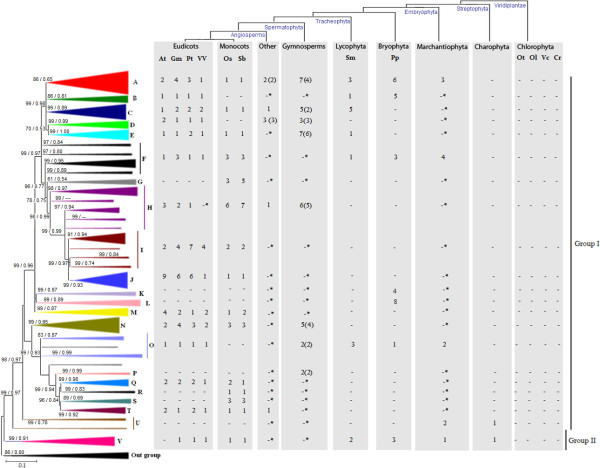
**Evolutionary profile of XTH in green plants**. Phylogenetic tree showing XTH PoGOs in green plants. The topology was obtained by Neighbor-Joining method with genetic distances calculated by p-distance. The bootstrap values higher than 50% are shown for 1000 replicates along with Maximum-likelihood (ML) aLRT values higher than 0.50 (in the figure [**NJ**/**ML**]). Each triangle represents a PoGO and the amount of sequences belonging to each PoGO is proportional to triangles' size. The number of genes by species is given (At - *Arabidopsis thaliana*, Gm *- Glycine max*, Pt - *Populus trichocarpa*, Vv - *Vitis vinifera*, Os - *Oryza sativa*, Sb *- Sorghum bicolor*, Sm - *Selaginella moellendorffii*, Pp - *Physcomitrella patens patens*, Ot - *Ostreococcus tauri*, Ol - *Ostreococcus lucimarinus*, Vc - *Volvox carteri*, Cr - *Chalmydomonas reinhardtii*). Outgroup is formed by fungus sequences. Accession numbers for all genes in the tree are described in Additional File 3 and the detailed tree is shown in Additional File 2. * - No genes found within a PoGO that lacks genomic information, only ESTs. ( ) - Given number of different species within a PoGO.

Two hundred and ninety-three XTH homologous sequences were identified among green plants. Phylogenetic analysis of these sequences resulted in 21 PoGOs and a group of paralogs among streptophytes (A to V in Figure 2; Additional File 2; Additional File 3). While no XTH sequence was found among the four complete genomes of green algae (chlorophytes), at least two land plant XTH founder genes were identified in the last common ancestor of streptophytes (PoGOs U and V in Figure 2). One of these genes was represented by *CvXTH1 *(previously identified as *Chara2 *[28]) from *Chara vulgaris *(class Charophyceae). This ancestral gene persisted in all streptophyte lineages and was represented by the single PoGO V (Figure 2). The other founder gene is represented by PoGO U (Figure 2), that integrates the *CpXTH1 *gene in the charophyte alga *Closterium peracerosum *(class Zygnemophyceae) and consisted exclusively of XTH sequences from marchantiophyte and streptophyte algae. PoGO U most likely gave rise to 19 PoGOs and a group of paralogs (group K in Figure 2). These PoGOs and paralogs included all previously reported *Arabidopsis*, rice, and poplar XTH genes (Figure 2; [6,29,30]). Since molecular and morphological data suggest that streptophyte algae are sister groups of land plants [31,32], and since liverworts such as *Marchantia *are the most basal embryophytes [33], it is plausible that PoGO U-related orthologs have been lost in bryophytes and tracheophytes (Figure 2). PoGO U and the homologous group that emerged from it were defined as Group I while the homologous genes from PoGO V were identified as Group II (Figure 2).

Together, these results indicate that PoGOs U and V share a common origin, which implies that the first XTH gene duplication and maintenance occurred before land colonization by plants. This conclusion is supported by the detection of XyG in all groups of land plants [7] and the presence of XyG transglycosylation activity in the charophyte alga *Chara vulgaris *[28]. Thus, XTH apparently originated after the divergence of chlorophyte and streptophyte algae. XyG was therefore probably absent in the more ancestral Viridiplantae lineages represented by chlorophyte algae and emerged as a new cell wall component in streptophytes. Since chlorophyte algae occur mainly in salt-water wheres streptophyte algae are mainly fresh-water, we suggest that XyG provided a selective advantage in the colonization of fresh-water habitats. In addition, the ability of XyG to confer mechanical strength [20] may have been particularly advantageous in allowing streptophytes to colonize terrestrial habitats. Successful land colonization by plants has apparently been limited to a sister lineage of streptophyte algae that gave rise to all embryophyte groups [32]. These conclusions provide additional support for the suggestion that the acquisition of XyG by streptophyte algae was an important factor in land colonization [34].

The PoGOs in Group I consisted of genes from all major embryophyte lineages. PoGOs A, F, and O that contained XTH genes from the marchantiophyte *Marchantia polymorpha *(liverwort) and PoGO B that contained bryophyte (moss) XTH genes appeared to have emerged from four genes in the last common ancestor of embryophytes (Figure 2). Thus, up to four ancestral XTH genes were related to early non-vascular land plants that were present at least 475 million years ago (based on the current fossil record) [35]. This conclusion supports the notion that gene duplication in the XTH family and its resulting selective advantages is an ancient phenomenon among land plant lineages.

A striking feature of Group I was the extensive amplification of XTH genes among angiosperms, i.e., 33 genes in *Arabidopsis*, 34 in soybean, 35 in poplar, 19 in grape, 30 in rice, and 32 in sorghum, all of which were distributed among 17 PoGOs (Figure 2). However, differential patterns of amplification and/or gene losses were observed among land plants. For instance, PoGOs B, D, and O were present among eudicots but were not detected in monocots (Figure 2). The presence of bryophyte and lycophyte XTH genes in PoGO B, gymnosperm XTHs in PoGO D, and embryophyte XTH genes in PoGO O indicates that gene losses from these PoGOs occurred specifically in the monocot lineage (Figure 2). On the other hand, PoGOs G, R, and S were restricted to monocots (Figure 2), which suggests that these groups emerged after the divergence of eudicots and monocots. Although the abundance of XTH genes in the rice genome [30] was initially considered unusual because of the small content of XyG in the PCW of most grasses [4,14], XyG can account for up to 10% of the wall mass in grass tissues during growth [36] and XTH activity may be more important for grasses than previously thought [30,37].

PoGO L and the set of paralogs genes that formed Group K were restricted to bryophytes whereas PoGO P was restricted to gymnosperms. The simplest explanation for this is that these lineage-specific acquisitions may be related to functional specialization and/or novelties. These lineage-specific differences suggest that distinct patterns of selective pressure acted on the XTH genes in different lineages, and may partly explain the differential abundance of XyG, i.e., 10-20% of the PCW dry weight in eudicots compared to <5% in graminaceous monocots [4,14,38,39]) and the different patterns of XyG substitution and structure, e.g., presence of galacturonic acid in bryophyte and marchantiophyte XyG [7].

PoGO N contained *Arabidopsis *proteins encoded by *At-XTH31 *and *At-XTH32*, which are involved exclusively in XyG hydrolysis and lack transglycosylation activity [6]. XTH with exclusively hydrolytic activity may have derived from transglycosylating proteins as a new feature of angiosperms [6]. However, this conclusion may need to be reevaluated in the light of the data presented here. Indeed, sequences from several taxonomic groups of flowerless seed plants (pinophytes, gnetophytes and cycadophytes; Figure 2) were included in PoGO L, suggesting that XTHs with exclusively hydrolytic activity emerged at least in the last common ancestor of the spermatophytes. This inference is further supported by the occurrence of hydrolytic activity in fast growing tissues such as meristems (*Arabidopsis*; [40]) or during specific developmental stages such as germinating seeds (tomato; [41]) or in physiological processes such as the mobilization of endosperm reserves (*Hymenaea courbaril*; [17,42]), all of which are key phenomena in seed plants and originated at least 300 million years ago, as suggested by the cycadophytes fossil record [43].

The moss *Physcomitrella *genome contained 30 XTH genes, a number comparable to that found in angiosperms (33 in *Arabidopsis *and 30 in rice). This elevated number of genes may reflect lineage-specific genome duplications in mosses [44]. Of these 30 genes, 27 were classified in Group I and three in Group II (Figure 2), and could be divided into six PoGOs and a paralog group (Group K, Figure 2). Two of these groups, PoGO L and the paralog group K, were bryophyte-specific while the other five PoGOs (A, B, F, O and V) were shared by tracheophytes (Figure 2; Additional File 3). The emergence of these lineage-specific XTH genes in bryophytes could be related to the presence of a specific type of XyG containing β-D-galactosyluronic acid and a branched xylosyl residue [7] that is not shared with tracheophytes [7,37].

The vascular seedless *Selaginella *had only 16 XTH genes (14 in Group I and two in Group II) that were divided into seven PoGOs conserved among other tracheophyte lineages (Figure 2). All *Selaginella *XTH genes occurred in PoGOs shared by angiosperms. The retention of these genes by angiosperms suggests that the early set of tracheophyte XTH genes was conserved in higher taxa, whereas the basic XyG pattern of XXXG emerged in hornworts and is shared by all tracheophytes [7,37]. The appearance of PoGOs C and E in *Selaginella *suggested that the last common ancestor of tracheophytes carried at least two additional XTH genes when compared to the last common ancestor of embryophytes (Figure 2). If each PoGO shared between different lineages is considered to be representative of founder genes then during their evolution the number of green plant XTHs gradually expanded from two ancestral genes in streptophyte algae to five in early embryophytes (*Physcomitrella *- Bryophyta), seven in early tracheophytes (*Selaginella *- Lycophyta), and 18 in angiosperms (eudicots and monocots). This increasing number of PoGOs suggests an important role for XyG in the evolution from non-vascular land plants to angiosperms.

The genes in PoGO V have not previously been reported to be XTH, perhaps because the model plant *Arabidopsis *lacks genes in this PoGO. Indeed, PoGO V included genes from all other complete embryophyte genomes and also *M. polymorpha *and the charophyte alga *C. vulgaris *(Figure 2; Additional File 3). A *C. vulgaris *cDNA sequence encoding a protein encompassing the main XTH catalytic site (DEIDFEFLG) has been isolated and may correspond to the XyG transglycosylation activity identified in growing tissues of this alga [28]. As in angiosperms, the *C. vulgaris *transglycosylase activity may be involved in adjustment of the PCW during growth [28].

We also searched for genes similar to XTH in animal and fungus genomes. Although no proteins similar to XTH were identified in the animal genomes, we found three glycosyl hydrolases in the complete *Saccharomyces cerevisiae *genome (*Utr2 *- NP_010874, *Crr1p *- NP_013314 and *Crh1p *- NP_011705; GenBank) and five in *Aspergillus nidulans *(XP_662119, XP_664552, XP_660657, XP_658537 and XP_661518; GenBank) that were similar to plant XTHs (Additional File 2). When these fungus sequences were analyzed together with those for XTHs from all groups of plants they formed an outgroup (Figure 2). The high bootstrap support (86%) suggested a possible single origin for these fungi hydrolases. However, it is unclear whether these fungus genes share a common ancestor with streptophytes XTHs or whether the similarity merely reflects functional convergence from an ancestral eukaryotic glycosyl hydrolase.

### β-Galactosidase genes are present in eukaryotes and were notably amplified during the evolution of land plants

**Figure 3:**
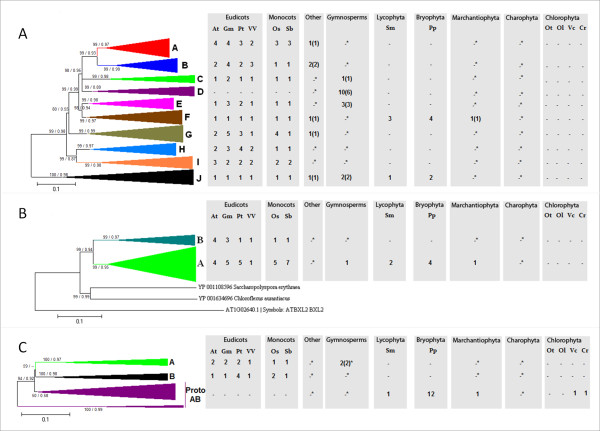
**Evolutionary profiles of β-galactosidase (A), XyG-active β-glucosidase (B) and α-xylosidase (C) genes in green plants**. The topologies were obtained by Neighbor-Joining method with genetic distances calculated by p-distance. The bootstrap values higher than 50% are shown for 1000 replicates along with Maximum-likelihood (ML) aLRT values higher than 0.50 (in the figure [**NJ**/**ML**]). Accession numbers for all genes in the trees are described in Additional File 5 for β-galactosidase, Additional File 7 for β-glucosidase and Additional File 9 for α-xylosidase. The detailed trees are shown in Additional File 4 for β-galactosidase, Additional File 6 for β-glucosidase and Additional File 8 for α-xylosidase. The tree containing the 100 first blastp hits of *AtXYL1 *is shown in Additional File 10.

One hundred and thirty-three non-redundant β-galactosidase genes were identified in the embryophyte lineages analyzed. These β-galactosidase genes were organized into 10 PoGOs that were divided into two homologous groups (Figure 3A; Additional File 4). Group I contained only PoGO J composed of animal, plant and fungus genes (Figure 3A; Additional File 5). On the other hand, Group II contained nine PoGOs present exclusively in plants (Figure 3A; Additional File 5). The presence in PoGO J of genes from all major eukaryotic lineages suggested that the plant-specific β-galactosidases in Group II must have derived from PoGO J after the divergence of plants from the fungus/animal lineage. PoGO J can therefore be considered to be representative of the ancestral β-galactosidase gene. Remarkably, all embryophytes had a single gene, except for *Physcomitrella*, which had two, possibly because of moss-specific genome duplication (Figure 3A; Additional File 5). In contrast, the β-galactosidases genes of Group II showed significant duplication events during plant evolution. β-Galactosidases from the non-vascular plants *Physcomitrella *(a bryophyte) and *Marchantia *(a marchantiophyte) and the vascular seedless *Selaginella *were restricted to PoGO F, indicating that this PoGO most probably emerged from PoGO J (Group I) genes in the last common ancestor of embryophytes (Figure 3A).

PoGOs C and E formed part of the β-galactosidase genes from gymnosperms and angiosperms, indicating that these PoGOs probably emerged from PoGO F in the last common ancestor of spermatophytes. PoGOs A, B, G, H, and I apparently emerged exclusively in angiosperms (Figure 3A; Additional File 5). However, the lack of a complete genome for gymnosperms means that the presence of genes belonging to this series of PoGOs in flowerless seed plants cannot be discarded.

PoGO D consisted exclusively of gymnosperm EST sequences from pinophytes, gnetophytes, and ginkgophytes. Based on the tree topology, the PoGO D genes probably emerged from PoGO C after the divergence of angiosperms and gymnosperms (Figure 3A). An alternative hypothesis is that these genes were selectively lost in the angiosperm lineage.

No β-galactosidase-coding genes were detected in Chlorophyta algae. This situation may reflect the loss of these genes from the *Chlamydomonas*, *Volvox*, *Ostreococcus tauri *and *O. lucimarinus *genomes, possibly because of the lack of selective pressure to maintain these enzymes.

### Land plant β-glucosidases active on XyG form two PoGOs: an ancestral one shared by all embryophytes and a derived one restricted to angiosperms

By using the protein sequence of the well characterized *Tropaeolum majus *(eudicot) β-glucosidase (CAA07070 - GenBank; [45]) as a query in our pipeline (Additional File 1) we identified 53 non-redundant plant sequences that were classified into two PoGOs (A and B; Figure 3B; Additional File 6; Additional File 7). PoGO A included the *Tropaeolum *β-glucosidase and genes from all major embryophyte lineages (Figure 3B). PoGO A also contained four *Arabidopsis *paralog genes (Additional File 7) coding for proteins characterized as XyG hydrolytic β-glucosidases present in apoplastic fluid [46]. The genes in PoGO B probably emerged from PoGO A and were detected exclusively among angiosperms (Figure 3B). *Arabidopsis *had four paralogs genes in PoGO B (Additional File 7) that are arranged *in tandem *in chromosome 3 whereas rice had a single gene (Additional File 7). Functional characterization of the PoGO B genes is still lacking.

No gene similar to XyG-active β-glucosidase was detected in the genomes of green algae, fungi or animals. Surprisingly, we found very similar sequences in some bacterial species. However, phylogenetic analysis of the evolutionary relationship between our set of plant β-glucosidases and the two most similar bacterial sequences (YP_001634696 from *Chloroflexus aurantiacus *and YP_001108596 from *Saccharopolyspora erythraea*; GenBank; Additional File 6) was inconclusive, although it is possible that these bacterial genes may share a common origin with plant XyG-active β-glucosidases. Several explanations could account for this scenario. First, the ancestral genes may have survived only in bacteria and streptophytes, having been lost in the fungal/metazoan group and in Viridiplante from chlorophytes. Second, these β-glucosidases could have a bacterial origin and were transmitted horizontally from the ancestral cyanobacterial endosymbiont, which gave rise to chloroplasts, to earlier Viridiplantae, but were specifically lost in chlorophytes. Third, the similarity between the plant XyG-active β-glucosidase and bacterial genes may simply be a case of convergent evolution from distinct ancestral hydrolases.

### Plant α-xylosidase emerged before the divergence between chlorophyte and streptophyte algae and is evolutionarily related to eukaryote α-glucosidases

At least 24 sequences significantly similar to the well-characterized α-xylosidase *AtXYL1 *from *Arabidopsis *(*At1g68560*; [47]) and *Tropaeolum majus *(CAA10382 - GenBank; [48]) were identified in the Viridiplantae species (Figure 3C). These genes were grouped into a single set of homologous sequences that were further organized into three PoGOs, of which PoGO A was spermatophyte-specific and PoGO B was restricted to angiosperms (Figure 3C; Additional File 8; Additional File 9). These two PoGOs probably emerged from PoGO Proto AB (Figure 3C; Additional File 8; Additional File 9) that includes genes from more ancestral land plants such as *Physcomitrella *(seven genes), and *Selaginella *(one gene) (Additional File 9). Finally, PoGO Proto AB included genes from the green algae *Chlamydomonas *and *Volvox *(Figure 3C; Additional File 8; Additional File 9). Unexpectedly, the Prasinophyceae algae *Ostreococcus tauri *and *O. lucimarinus *had no genes in PoGO Proto AB, suggesting that α-xylosidase genes were specifically lost in these organisms after their divergence from other Viridiplantae lineages.

An interesting feature of α-xylosidases was the extensive gene duplication in the *Physcomitrella *genome, which contained at least 12 genes compared to three in *Arabidopsis *and a single gene in *Selaginella *(Figure 3C; Additional File 9). This greater number of genes suggests that α-xylosidase gene duplication and fixation in these basal embryophytes may have conferred some selective advantage possibly related to the ecological role of mosses. A plausible explanation for the evolutionary development of α-xylosidase could be that a single ancestral gene in green algae (represented by PoGO Proto AB) eventually gave rise to spermatophyte-specific PoGO A and angiosperm-specific PoGO B (Figure 3C).

To improve our understanding of the origin of plant α-xylosidases, we extended our analysis to all genes that shared any similarity with the query sequences from *Arabidopsis *and *Tropaeolum *(e-value < e^-4^), including sequences obtained from searches against bacteria and the fungal/metazoan group (Additional File 10). In *Arabidopsis*, the genes most closely related to α-xylosidases were α-glucosidases *RSW3 *(radial swelling 3; At5g63840), which shared 27% identity with *AtXYL1 *(245 out of 903 amino acids; e-value = 1e-^-81^), and *HGL1 *(heteroglycan glucosidase 1; AT3G23640), which shared 33% identity with *AtXTYL1 *(185 out of 559 amino acids; e-value = 6e^-79^). A phylogenetic analysis that integrated α-xylosidase homologues and *RSW3 *and *HGL1 *homologues from green plants with the most closely related corresponding sequences from fungi, animals and bacteria (Additional File 10) showed that the *RSW3 *and *HGL1 *genes formed a single PoGO within plants. The *HGL1 *PoGO included genes from embryophytes and the most similar non-plant sequences were from bacteria. No genes from fungi or animals were included in the *HGL1 *PoGO. The *RSW3 *PoGO included genes from all Viridiplantae lineages, including the Prasinophyceae algae *O. tauri *and *O. lucimarinus*. *rsw3 *is a temperature-sensitive mutant of *Arabidopsis *that has radially swollen roots and a deficiency in cellulose deposition. *RSW3 *is thought to process N-linked glycans in the endoplasmatic reticulum, as part of the quality control pathway to ensure correct protein folding [49]. In our analysis, the *RSW3 *gene shared high similarity with the catalytic α-subunit of fungal and animal glucosidase II. Together, these findings suggest that the plant-specific α-xylosidase involved in XyG mobilization evolved from an ancestral eukaryotic α-glucosidase gene, represented here by *RSW3 *PoGO (Additional File 10). This finding also supports the idea that neofunctionalization could be the main process responsible for the switch in substrate specificity from α-glucosidase to α-xylosidase during the evolution of glycosyl hydrolases.

### XyG β-(1→4)-glucan synthase belongs to the celulose synthase-like C gene family present in streptophyte algae

**Figure 4:**
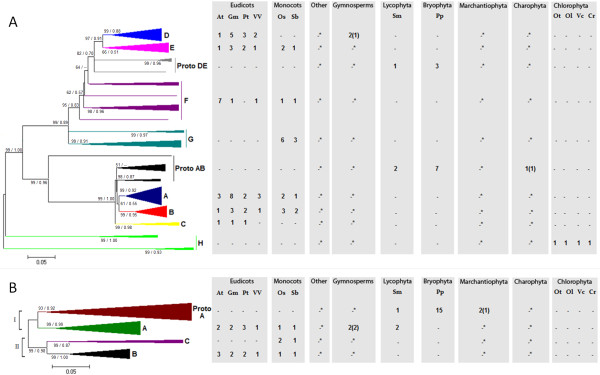
**Evolutionary profiles of β-glucan synthase (A) and XXT (B) genes in green plants**. A: CSL C (β-glucan synthase) and CSL A (β-mannan and β-glucomannan synthases) PoGOs. The topologies were obtained by Neighbor-Joining method with genetic distances calculated by p-distance. The bootstrap values higher than 50% are shown for 1000 replicates along with Maximum-likelihood (ML) aLRT values higher than 0.50 (in the figure [**NJ**/**ML**]). Accession numbers for all genes in the trees are described in Additional File 12 for β-Glucan Synthase and Additional File 14 for XXT. The detailed trees are shown in Additional File 11 for β-Glucan Synthase and Additional File 13 for XXT.

Ninety-one genes (threshold of e^-04^) related to the *Arabidopsis *celulose synthase-like (CSL) gene *AtCSLC4 *(*At3g28180*) encoding a β-(1→4)-glucan synthase [19] were identified by searching the Viridiplantae database. The threshold of e^-04 ^used to define significant similarity throughout this study allowed the recovery of genes that formed the CSL C [50,51] and CSL A [51] groups, as well as a group of chlorophyte genes that behaved as an outgroup to CSL C and CSL A in our analysis (Figure 4A; Additional File 11; Additional File 12). This finding supports the suggestion that CSL C and CSL A resulted from a duplication event of an ancestral green plant gene present in chlorophytes, and agrees with a recently published report [51]. This ancestral gene is represented in our analysis by PoGO H, which contained a single copy in each of the green algae genomes analyzed (*Volvox*, *Chlamydomonas*, *O. tauri *and *O. lucimarinus*; Figure 4A; Additional File 12).

Based on an analysis of complete genomes from land plants and chlorophyte algae, Yin *et al. *[51] concluded that the CSL C and CSL A groups were the products of an ancestral gene duplication in earlier embryophytes. This conclusion may have to be re-evaluated since, as shown here, CSL C included a gene from the streptophyte alga *Chara globularis *(Charophyta), indicating that this group emerged before land colonization by plants (Figure 4A). These data raise the interesting possibility that the gene duplication event that resulted in CSL A and CSL C had occurred in early streptophytes. A recent work [52] has detected a XyG-like polymer containing glucose and xylose in the streptophyte algae *Spirogyra *(Class Zygnematophyceae). In contrast, Popper and Fry [53] reported the absence of XyG in the cell walls of charophycean algae such as *Chara*, *Nitella*, *Coleochaete *and *Klebsormidium*. Although these partially contradictory results indicate that more research is needed to understand the composition of the PCW in streptophyte algae, it seems plausible that the CLS C gene from *C. globularis *could be involved in the synthesis of a XyG-like polymer, as occurs in *Spirogyra*.

Members of CLS A (*AtCSLA9 *[At5g03760 - PoGO D], *AtCSLA2 *[At5g22740 - PoGO E] and *AtCSLA7 *[At2g35650 - PoGO F]) have β-mannan synthase activity when expressed in S2 *Drosophila *cells supplied with GDP-mannose [54]. Interestingly, the proteins encoded by these three genes were also able to produce β-glucomannan when supplied with GDP-mannose and GDP-glucose, and unexpectedly AtCSLA9 (PoGO D) produced β-glucan when supplied with GDP-glucose; this latter activity is believed to be the main function of CSL C. Thus, it appears that CSL A can synthesize at least three different polymers, β-mannan, β-glucomannan and β-glucan. Conversely, to date, only a single activity (β-glucan synthesis) has been described for CSL C members. These observations raise the question of which activity is performed by the ancestral PoGO H members in chlorophytes. Only mannans, glucoronic acids, mannuronic acids and 3-O-methyl rhamnose have been detected in the cell walls of chlorophytes [55], thus supporting the view that β-mannan synthase is the basic or primordial activity of the plant CSL A and C ancestral group represented by chlorophyte PoGO H [37,51]. This conclusion further supports the hypothesis that the specific β-glucan synthase activity used in XyG synthesis emerged from mixed activity (β-mannan/β-glucomannan/β-glucan synthase) proteins in the course of streptophyte evolution.

The CSL C group included five genes from *Arabidopsis*, poplar and rice, 12 from soybean, four from grape, three from sorghum, two from *Selaginella *and seven from *Physcomitrella*. The group was further divided into four PoGOs (A, B, C, and Proto AB; Figure 4A). PoGOs A and B included angiosperm genes whereas PoGO C was restricted to eudicots. PoGO A included the *Arabidopsis AtCSLC4 *gene that was shown to encode a β-(1→4)-glucan synthase involved in XyG biosynthesis [19]. PoGO Proto AB included *Selaginella*, *Physcomitrella *and *C. globularis *genes, with the angiosperm PoGOs A and B probably resulting from the duplication of an original PoGO Proto AB gene within the angiosperm lineage.

The CSL A group included genes from all embryophyte lineages. The genes in this group were divided into five additional PoGOs (D, E, Proto DE, F and G; Figure 4A). PoGO D was spermatophyte-specific but lacked genes from monocots, which suggests these genes were specifically lost in the monocot lineage. PoGO D included *Arabidopsis AtCSLA9*, the protein product of which has important β-mannan synthase activity, as well as β-glucomannan and β-glucan synthase activities [54]. PoGO E, which is more closely related to PoGO D, was restricted to angiosperms and included *Arabidopsis AtCSLA2*, which has prevalent β-mannan synthase activity and β-glucomannan synthase activity, but almost no β-glucan synthase activity. PoGO Proto DE, which contained sequences from *Selaginella *and *Physcomitrella*, was considered as an outgroup to PoGOs D and E (Figure 4A). This finding suggested that PoGOs D, E and Proto DE had a common origin in the last common embryophyte ancestor. PoGO F was restricted to angiosperms and contained *Arabidopsis AtCSLA7*, which has mainly β-mannan synthase activity and lacks β-glucan synthase activity [54]. PoGO F had an apparent *Arabidopsis*-specific gene duplication pattern that resulted in seven paralogs whereas the grape, soybean, sorghum and rice genomes possess a single gene. The relevance of these lineage-specific gene duplication events remains to be investigated. Another striking feature of the CSL A group was the monocot-specific PoGO G.

Based on the evidence presented here, we conclude that XyG-specific β-glucan synthases in CSL C evolved from an ancestral β-mannan synthase represented by PoGO H, the ancestral group of the CSL C and A families. The presence of CSL C genes in charophytes is strong evidence that XyG emerged prior to the colonization of land by early embryophytes. This conclusion agrees with the recent detection of XyG in the cell walls of Charophycean algae [37].

### α-Xylosyltransferases (XXT) are present in all land plant lineages but absent from chlorophyte algae

An analysis of 45 XXT genes resulted in the recognition of two homologous groups among embryophyte XXTs (Groups I and II in Figure 4B). Group I was the most ancient and included PoGO Proto A that contained genes from *Marchantia *(Marchantiophyta), *Physcomitrella *(Bryophyta) and *Selaginella *(Lycophyta), and PoGO A that included *Selaginella*, gymnosperm and angiosperm genes (Figure 4B; Additional File 13; Additional File 14). We suggest that PoGO A emerged from Proto A by gene duplication in the last common ancestor of tracheophytes. PoGO A included the *Arabidopsis XXT1 *and *XXT2 *genes [20]. The *xxt1/xxt2 *double mutant lacks detectable XyG, but the only apparent phenotypes associated with these mutations were aberrant root hair development, slow growth, and a slightly smaller stature at maturity [20]. This result challenges the conventional model for the structure of the PCW in eudicot and non-graminaceous monocots, which states that XyG is the principal load-bearing structure [4,14,56-60]. In the light of this traditional PCW model a plant lacking XyG would not be viable or at least would have a very deleterious phenotype, which apparently is not the case, at least in *Arabidopsis*.

Group II contains *Arabidopsis XXT5 *(At1g74380), which participates in XyG synthesis [61]. *xxt5 *exhibits a phenotype similar to that described for the *xxt1*/*xxt2 *double mutant and consists of short root hairs with bubble-like extrusions at the tip. In addition, the main root cell morphology was altered in the *xxt5 *mutant and the level of XyG was reduced. Unexpectedly, although *XXT5 *was expressed in the *xxt1*/*xxt2 *double mutant no XyG was detected in these mutants, possibly indicating an epistatic effect whereby the activity of either *XXT1 *or *XXT2 *is required before *XXT5 *can act [20]. It will be interesting to evaluate whether the Group I XXTs of other species have this type of epistatic effect on genes in Group II.

### Two types of evolutionarily unrelated plant α-fucosidases are active against XyG oligosaccharides

**Figure 5:**
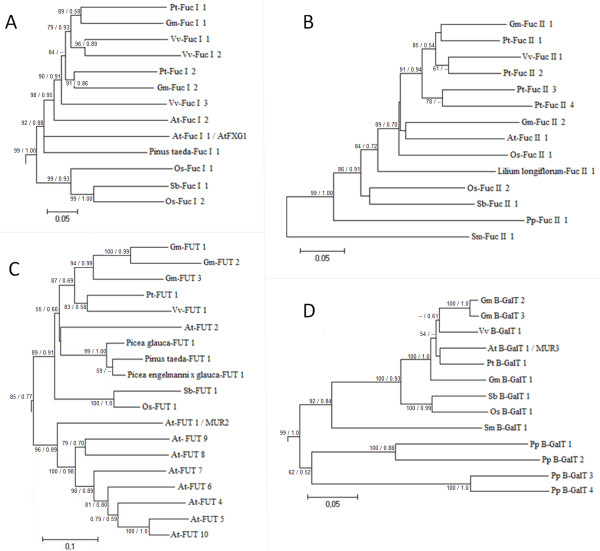
**Evolutionary profiles of α-fucosidase type I (A), α-fucosidase type II (B), α-fucosyltransferase (C) and β-galactosyltransferase (D) genes in green plants**. A: *Arabidopsis AtXYL1 *orthologs. B: *Lilium longiflorum EBM II *orthologs. C: *Arabidopsis Mur2 *orthologs. D: *Arabidopsis Mur3 *orthologs. The topology was obtained by Neighbor-Joining method with genetic distances calculated by p-distance. The bootstrap values higher than 50% are shown for 1000 replicates along with Maximum-likelihood (ML) aLRT values higher than 0.50 (in the figure [**NJ**/**ML**]). Accession numbers for all genes in the trees are described in the following Supplemental Tables: A and B - Additional File 16, C - Additional File 18 and 18D - Additional File 20. The detailed trees are shown in the following Additional Files: A - Additional File 15, C - Additional File 17 and 17D - Additional File 19.

Two genes were found to encode XyG-active α-fucosidases: the *Arabidopsis *gene *AtFXG1 *(At1g67830; [18]), which belongs to the largely unknown GDSL-motif lipase/hydrolase family protein (Figure 5A; Additional File 15; Additional File 16) and the *Lilium longiflorum *gene *EBM II *(BAF85832 - GenBank; [21]; Figure 5B; Additional File 16). Interestingly, these proteins shared no similarity with each other (e-value = 8.3) and were therefore evolutionarily unrelated but converged functionally to fulfill a similar enzymatic activity.

The *Arabidopsis *genes most closely related to *AtFXG1 *(Additional File 15; Additional File 16) lacked any functional information, and we will therefore focus here on the PoGO that included *AtFXG1 *(PoGO A; Figure 5A). PoGO A contained another *Arabidopsis *gene, *At3g26430*, and included genes from other angiosperms, as well as gymnosperm ESTs, which lead to the conclusion that this group emerged in the last common ancestor of the Spermatophyta. To extend our understanding of the evolutionary origin of *AtFXG1 *homologues in plants, we performed a broader phylogenetic analysis (Additional File 15) that encompassed the first 100 blast hits obtained with an *AtFXG1*-encoded protein sequence query run against the Viridiplantae 1.0 dataset using an e-value threshold of e^-4 ^(see Methods). This analysis identified PoGOs 1 and 2 that emerged before angiosperm divergence (Additional File 15). These two PoGOs included *Selaginella *(PoGOs 1 and 2) and *Physcomitrella *(PoGO 2) with angiosperm genes, which suggests that they emerged at least in the last common ancestor of tracheophytes and land plants, respectively. No gene similar to *AtFXG1 *was detected in chlorophytes, suggesting that hydrolases of this type were restricted to embryophyte lineages. Based on the tree topology shown in Additional File 15, it is likely that PoGO A XyG-active α-fucosidases emerged from PoGO α. The functional characterizations of genes from PoGOs α and β should improve our understanding of the diversification of α-fucosidase from GDSL-motif lipase/hydrolase family protein.

*Lilium longiflorum EBM II *homologues among green plants formed a single PoGO (PoGO B; Figure 5B; Additional File 16) that was unrelated to the GDSL-motif lipase/hydrolase gene family. PoGO B arose at least in the last common ancestor of embryophytes. *Arabidopsis *had a single gene in PoGO B (*At4g34260*) that was recently confirmed to encode a protein with XyG α-fucosidase activity (*AtFuc95A*; [62]). Green algae genomes contained no genes similar to PoGO B members, suggesting that α-fucosidases homologous to *EBM II *are limited to land plants, in a manner similar to *AtFXG1 *homologues.

### α-Fucosyltransferases orthologs to *Mur2 *are present among spermatophytes and share similarity with uncharacterized embryophyte genes

To determine the evolutionary profile of XyG α-fucosyltransferase in plants we used the protein encoded by the functionally characterized *Arabidopsis Mur2 *gene (At2g03220; [63]) as a query. This strategy identified 82 possible *Mur2 *homologous genes among embryophytes (Additional File 17). Because there is little functional information for this family, we will limit our discussion to PoGO A, which contains *Mur2 *and includes genes from angiosperms and gymnosperms (Figure 5C; Additional File 18).

The presence of gymnosperm sequences suggested that PoGO A must have emerged in the last common ancestor of spermatophytes. This finding agreed with the presence of fucosylated XyG exclusively among spermatophytes [55]. Since the *Arabidopsis mur2 *mutant contains <2% of wild-type fucosylated XyG [63] it is likely that the protein encoded by *Mur2 *is the principle activity responsible for the transfer of fucosyl residues to XyG. In contrast, the *Arabidopsis *genome contains another set of nine genes that share high similarity with *Mur2 *(Additional File 17), of which eight paralogs are present in PoGO A (Figure 5C; Additional File 18). The role played by these genes remains unclear and it will be interesting to understand the genetic interaction between these genes and *Mur2*.

### β-Galactosyltransferases emerged in early land plants and share similarity with an extensive group of poorly characterized genes in green plants

The protein sequence of the well characterized β-galactosyltransferase gene *Mur3 *(At2g20370) from *Arabidopsis *[64] was used as a query to search for homologous genes among green plants. Madson *et al. *[64] showed that *Mur3 *has sequence similarity to animal exostosins, which are proteins involved in biosynthesis of the extracellular matrix. Our search revealed 191 genes that were possibly homologous to *Mur3*, none of which has been functionally characterized (Additional File 19). Within this extensive group of genes, several from chlorophytes (27 from *Chlamydomonas*, 16 from *Volvox*, three from *O. tauri *and two from *O. lucimarinus*) could represent the ancestral exostosin-like genes in plants (Additional File 19) from which the XyG galactosyltranferase activity probably evolved.

Functional information about this large family is restricted to *Mur3*, which is included in the embryophyte-specific PoGO A (Figure 5D; Additional File 20). *Mur3*-encoded protein acts specifically on the third xylose residue in the XXXG core structure of XyG, implying that other related enzymes transfer the galactosyl residues to the second xylose residue [64]. The candidate genes associated with the latter activity in *Arabidopsis *must be *At2g29040*, *At4g13990 *and *At2g32750*, which are the genes most closely related to *Mur3 *(Figure 5D).

## Conclusions

The comparative genomic analysis of enzymes involved in XyG synthesis and turnover described here has provided a few key conclusions about the evolution of this polymer in green plants. Evidence from non-XyG-bearing chlorophyte and streptophyte green algae indicates that part of the embryophyte XyG-related machinery (XTH, β-[1→4]-glucan synthase from the CSL C family and α-xylosidase) evolved in an aquatic environment, before land colonization by plants. This conclusion agrees with a recent report by Sørensen *et al. *[37] who used a combination of monosaccharide linkage analysis, CoMPP and immunolabeling to detect XyG in the PCW of some Charophycean algae, including Charales, Coleochaetales, and Zygnematales. Although Popper and Fry [53] detected no XyG in the cell wall of streptophyte algae such as *Chara*, *Nitella*, *Coleochaete *and *Klebsormidium*, the presence of an XyG-like polymer containing glucose and xylose was also reported in the alga *Spirogyra *(a streptophyte from the Class Zygnematophyceaes; [52]). In addition, XyG endotransglycosylase (XTH) activity has been detected in growing tissues of *Chara *[28]. Together, these observations suggest that in Charophycean algae a XyG-like polymer may be part of the PCW structure and that the mechanism by which hemicellulose is transglycosylated to adapt the PCW to cellular growth is conserved among streptophytes.

**Figure 6:**
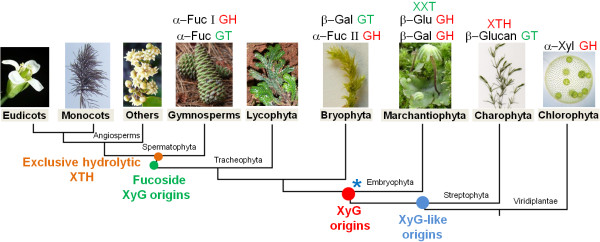
**Evolutionary model of XyG-related genes emergence in Viridiplantae kingdom**. The model shows the ancient origins we could trace back of each XyG-related gene families and the major events of XyG evolution in plants. XyG-like (containing only glucose and xylose) emerged in streptophytes algae, XyG (containing glucose, xylose, and galactose) emerged in early embryophytes and the fucosylated XyG emerged in the last common ancestor of spermatophytes. (*) indicates the possible origins of the ancestral genes that gave rise to Spermatophytes α-Fucosyl Transferase (*Mur2 *orthologs) and α-Fucosylase type I (*AtXYL1 *orthologs). GH - Glycosyl Hydrolases and GT - Glycosyl Transferases.

Streptophyta algae have at least three enzymes involved in XyG synthesis and turnover that are homologous to those of embryophytes, namely, XTH, β-(1→4)-glucan synthase and α-xylosidase (Figure 6). Homologous of α-xylosidase are present in Chlorophyta algae that completely lack XyG. Overall, our findings support the idea that a primordial XyG-like polymer emerged before land colonization by plants. The selective advantage conferred by this polymer may have been related to cell-cell attachment features within streptophytes multicellular algae rather than to mechanical structure [52]. Once the land was colonized, XyG was definitively incorporated into the PCW, as exemplified by the presence of XyG in basal land plants [7].

Our evolutionary data highlight the great functional plasticity of XyG glycosyl hydrolases (GHs) and XyG glycosyl transferase (GTs) in the course of green plant evolution. For example, α-xylosidase activity possibly emerged from α-glucosidase, β-(1→4)-glucan synthase-specific enzymes possibly emerged from enzymes with β-mannan activity, and α-fucosidase type I possibly emerged from the GDSL-motif lipase/hydrolase family.

There was a positive correlation between the number of founder genes in XyG-related gene families, defined by the number of PoGOs, and the growing complexity of the PCW. For instance, the number of PoGOs involving XTH sequences from streptophyte algae was limited to two but increased to 12 PoGOs shared amongst eudicots and monocots (Figure 2). Overall, the higher number of PoGOs found to include angiosperm XyG-related genes compared to other plant groups was probably related to the high degree of specialization (expression pattern and/or functional novelties) among gene copies in angiosperm species.

In contrast, there was no clear correlation between the gene copy numbers of XyG GHs and GTs and the amount of XyG in PCW. For example, the moss *Physcomitrella*, as well as rice and sorghum (i.e, graminaceous monocots), in which XyG accounts for <5% of the PCW dry-weight, had 30, 30 and 32 XTH genes, respectively, a number similar to that observed in eudicots (33 in *Arabidopsis*, 35 in poplar and soybean) in which XyG accounts for 10-20% of the PCW dry-weight. We speculate that remodeling of the PCW by the selective turnover and transglycosylation of XyG may be important, even for species with low amounts of XyG.

The role of XyG in embryophyte PCW remains unclear, although recent work has shown that *Arabidopsis *mutant plants with undetectable XyG have an almost normal development [20]. More research is needed to improve our knowledge of the mechanical structure of the PCW and the relationships among its components.

Our data suggest that the colonization of land by plants was marked by a notable increase in the sophistication of the machinery required for XyG biosynthesis and turnover when compared to the pathways present in Streptophyta algae. This finding suggests that complex systems involving several enzymes may evolve in a stepwise manner, with each new step providing some selective advantage, as seen in the galactosylation of embryophyte XyG and fucosylation of spermatophyte XyG (Figure 6). XTHs with exclusively hydrolytic activity emerged by the neofunctionalization of an enzyme with mixed activity (transglycosylase/hydrolase) in the last common ancestor of spermatophytes. Finally, our data provide additional evidence to support the idea that Streptophyta algae and land plants are sister groups because they share XyG-related enzymes (XTH and CLS C) that are not present in chlorophytes (Figure 6).

## Methods

### Green plant sequence datasets

We generated a dataset of green plant proteins (Viridiplantae 1.0) that included 365,187 protein sequences from several completed genomes (*Arabidopsis thaliana*, version 7.0 - http://www.arabidopsis.org; *Populus trichocarpa*, version 1.1 - http://genome.jgi-psf.org/poplar/poplar.home.html; *Glycine max*, version 0.1 - http://www.phytozome.net/soybean.php; *Oryza sativa*, version 5.0 - http://www.tigr.org/tdb/e2k1/osa1/pseudomolecules/info.shtml; *Sorghum bicolor*, version 1.4 - http://genome.jgi-psf.org/Sorbi1/Sorbi1.home.html; *Selaginella moellendorffii*, version 1.0 - http://genome.jgi-psf.org/Selmo1/Selmo1.home.html; *Physcomitrella patens patens*, version 1.1 - http://genome.jgi-psf.org/Phypa1_1/Phypa1_1.home.html; *Volvox carteri*, version 1.0 - http://genome.jgi-psf.org/Volca1/Volca1.home.html; *Chlamydomonas reinhardtii*, version 3.0 - http://genome.jgi-psf.org/chlre3/chlre3.home.html; *Ostreococcus lucimarinus*, version 2.0 - http://genome.jgi-psf.org/Ost9901_3/Ost9901_3.home.html and *Ostreococcus tauri*, version 2.0 - http://genome.jgi-psf.org/Ostta4/Ostta4.home.html.

In addition, 402,770 assembled ESTs from *Adiantum capillus-veneris *(7,715), *Amborella trichopoda *(6,649), *Ceratodon purpureus *(1,044), *Ceratopteris richardii *(4,492), *Chamaecyparis obtusa *(4,061), *Closterium peracerosum *(1,716), *Cryptomeria japonica *(9,098), *Cycas rumphii *(4,335), *Ginkgo biloba *(4,178), *Gossypium hirsutum *(70,667), *Liriodendron tulipifera *(7,087), *Marchantia polymorpha *(10,721), *Nuphar advena *(8,144), *Persea americana *(6,700), *Physcomitrella patens patens *(45,149), *Picea abies *(5,204), *Picea engelmannii × glauca *(14,201), *Picea glauca *(49,412), *Picea sitchensis *(25,425), *Pinus pinaster *(13,067), *Pinus taeda *(78,873), *Populus trichocarpa *(31,082), *Pseudotsuga menzienssi *(12,074), *Saruma henryi *(6,956), *Selaginella lepidophylla *(2,861), *Taiwania cryptomerioides *(778), *Tortula ruralis *(7,689) and *Welwitschia mirabilis *(6,680) were downloaded from the TIGR Plant Transcript Assemblies Database [65] and pooled to form the ViridiEST 1.0 database.

### Identification of XyG-related genes

To identify XTH, α-xylosidase, β-galactosidase, β-glucosidase, α-fucosidase, β-(1→4)-glucan synthase, α-fucosyltransferases, β-galactosyltransferases and XXT we ran Blast [66] searches against Viridiplantae 1.0 and ViridiEST 1.0. We also performed online Blast searches against the genomic sequences of complete plant genomes using the NCBI http://www.ncbi.nlm.nih.gov/, Phytozome http://www.phytozome.net/ and JGI Eukaryotic Genomes http://genome.jgi-psf.org/ databanks to ensure an exhaustive search. However, no protein sequences were incorporated from the genomic sequence searches if they were not already present in predicted proteomes of the different genome initiatives.

The amino acid sequences of previously reported genes were used as queries. For XTH queries we used 33 *A. thaliana *[29] and 29 *O. sativa *[30] proteins. For β-galactosidase we used 17 *A. thaliana *and 15 *O. sativa *proteins [67]. For XyG-active β-glucosidase we used the well-characterized *Tropaeolum majus *β-glucosidase (CAA07070 - GeneBank; [45]) as the query. For α-xylosidases we used queries from *A. thaliana *(*AtXYL1*, At1g68560 - TAIR; [47]) and *T. majus *(CAA10382 - GeneBank; [48]). For α-fucosidase we used the protein sequences encoded by *AtFXG1 *(At1g67830 - TAIR; [18]) and *EBM II *(BAF85832 - GeneBank; [21]) from *A. thaliana *and *Lilium longiflorum*, respectively. For β-(1→4)-glucan synthase we used the protein sequence encoded by *CSLC4 *from *A. thaliana *[19]. For α-fucosyltransferases the query was the *AtFUT1 *(*Mur2 *[63]). For β-galactosyltransferases we used the protein encoded by *Mur3 *(At2g20370) from *Arabidopsis *as the query [68]. For XXT we used the *Arabidopsis *genes *XXT1 *(At3g62720; [20]), *XXT2 *(At4g02500; [20]) and *XXT5 *[61].

The complete bioinformatics pipeline that was designed to perform similarity searches and used to produce non-redundant nucleotide and amino acid sequence data-sets is detailed in Additional File 1. We developed two programs used in the pipeline: BTF ("Blast to Fasta") and ETTool ("ESTs Translator Tool"). BTF reads the Blast results, and places the resulting subjects in a Fasta file. ETTool reads the tblastn results of protein queries against EST databases and selects only the blocks of amino acids that aligned between the queries and EST subjects; these blocks were transferred to a Fasta file.

### Phylogenetic analysis

The amino acids sequences were aligned with ClustalW [69] using the default parameters and then adjusted manually. All phylogenetic analyses were done using MEGA4.0 [70]. Phylogenetic distance tree topologies were obtained by the neighbor-joining method [26] with distances calculated by the PAM 001 distance matrix [25] and p-distances using 1000 bootstrap replicates. Maximum likelihood analyses were done in PhyML 3.0 [27] using the LG substitution model and an LTR statistical test [27]. All sequences used in this study are available upon request.

### Identification of possible groups of orthologs (PoGOs)

The detailed evolutionary analysis of XyG-related gene families allowed the identification of PoGOs. A PoGO was defined by the following criteria: (1) members of a PoGO were assumed to have a monophyletic origin, indicated by a bootstrap support greater than 50%; (2) a PoGO possessed at least one representative gene from *A. thaliana *and/or *O. sativa*, assuming that the putative complete set of genes for these organisms had been identified. In the case of a PoGO being restricted to some lineage, e.g., mosses or gymnosperms, the presence of sequences from at least two species of the same lineage in this PoGO was required.; and (3) the inferred phylogeny should be consistent with the known phylogeny of plant species [71].

## Abbreviations

CSL: cellulose synthase-like; ESTs: expressed sequence tags; GH: glycosyl hydrolase; GT: glycosyl transferase; ML: maximum likelihood; NJ: neighbor joining; PCW: primary cell wall; PoGO: possible group of orthologs; XTH: xyloglucan transglycosylase/hydrolase; XyG: xyloglucan; XXT: α-xylosyl transferase.

## Authors' contributions

LEVDB idealized the research, performed all the analysis and wrote the manuscript. MGAV is a group leader, has intellectual input in all presented results and conclusions and manuscript elaboration. Both authors read and approved the final manuscript.

## Supplementary Material

Additional file 1 **Bioinformatics search protocol**. The pipeline was used in construction of non-redundant protein data-sets. The arrows with asterisks represent manually conducted processes. The e-value cutoffs were 1e^-4 ^for Blastp and tBlastn. Our own programs (BTF and ETTool, both written in JAVA^®^) were developed for this protocol (available upon request). False positives from B1 and B2 protein sets were eliminated from the alignment by visual confrontation with reference sequences. A NJ tree was generated using B3 and B4 sets together with reference sequences. Redundant sequences and alternative splicing isoforms were eliminated by manual inspection of resulting tree. The final non-redundant protein data-sets obtained were used in our analyses. Click here for file

Additional file 2 **Detailed phylogenetic analysis of XTH gene family in green plants**. PoGOs names and color scheme are the same of Figure 2. The topology was inferred by Neighbor-Joining (NJ) method with 1000 bootstraps replicates and the genetic distances were calculated using p-distance. Bootstrap values higher than 50% are shown. Click here for file

Additional file 3 **XTH Possible Groups of Orthologs (PoGOs) in green plants**. Classification of XTH PoGOs by taxonomic ranking and the complete list of gene IDs. Click here for file

Additional file 4 **Detailed phylogenetic analysis of β-galactosidase gene family in green plants**. PoGOs names and color scheme are the same of Figure 3A. The topology was inferred by NJ method with 1000 bootstraps replicates and the genetic distances were calculated using p-distance. Bootstrap values higher than 50% are shown. Click here for file

Additional file 5 **β-galactosidase Possible Groups of Orthologs (PoGOs) in green plants**. Classification of β-galactosidase PoGOs by taxonomic ranking and the complete list of gene IDs. Click here for file

Additional file 6 **Detailed phylogenetic analysis of β-glucosidase gene family in green plants**. Description: PoGOs names and color scheme are the same of Figure 3B. The topology was inferred by NJ method with 1000 bootstraps replicates and the genetic distances were calculated using p-distance. Bootstrap values higher than 50% are shown. Click here for file

Additional file 7 **β-glucosidase Possible Groups of Orthologs (PoGOs) in green plants**. Classification of β-glucosidase PoGOs by taxonomic ranking and the complete list of gene IDs. Click here for file

Additional file 8 **Detailed phylogenetic analysis of α-xylosidase gene family in green plants**. PoGOs names and color scheme are the same of Figure 3C. The topology was inferred by NJ method with 1000 bootstraps replicates and the genetic distances were calculated using p-distance. Bootstrap values higher than 50% are shown. Click here for file

Additional file 9 **α-xylosidase Possible Groups of Orthologs (PoGOs) in green plants**. Classification of α-xylosidase PoGOs by taxonomic ranking and the complete list of gene IDs. Click here for file

Additional file 10 **Phylogenetic analysis of α-xylosidase related homologous groups in Eukaryotes and Bacteria**. All sequences analyzed were selected using *AtXYL1 *from *Arabidopsis *(*At1g68560*) as query in blast searches with e-value cutoff of e^-4^. The topology was inferred by NJ method with 1000 bootstraps replicates and the genetic distances were calculated using p-distance. Bootstrap values higher than 50% are shown. Click here for file

Additional file 11 **Detailed phylogenetic analyses of CSL-A and CSL-C (β-Glucan Synthase) gene families in green plants**. PoGOs names and color scheme are the same of Figure 4A. The topology was inferred by NJ method with 1000 bootstraps replicates and the genetic distances were calculated using p-distance. Bootstrap values higher than 50% are shown. Click here for file

Additional file 12 **CSL C (β-glucan synthase) and CSL A Possible Groups of Orthologs (PoGOs) in green plants**. Classification of CSL C and A PoGOs by taxonomic ranking and the complete list of gene IDs. Click here for file

Additional file 13 **Detailed phylogenetic analysis of α-xylosyl transferase (XXT) gene family in green plants**. PoGOs names and color scheme are the same of Figure 4B. The topology was inferred by NJ method with 1000 bootstraps replicates and the genetic distances were calculated using p-distance. Bootstrap values higher than 50% are shown. Click here for file

Additional file 14 **XXT Possible Groups of Orthologs (PoGOs) in green plants**. Classification of XXT PoGOs by taxonomic ranking and the complete list of gene IDs. Click here for file

Additional file 15 **Detailed phylogenetic analysis of α-fucosidase type I gene family in green plants**. *AtFXG1 *(At1g67830) PoGO is marked (Figure 5A). The topology was inferred by NJ method with 1000 bootstraps replicates and the genetic distances were calculated using p-distance. Bootstrap values higher than 50% are shown. This analysis allowed identification of PoGOs 1 and 2 which integrate *Selaginella *and *Physcomitrella *genes, suggesting that they emerged at least in the last common ancestor of land plants and represent the ancestral groups. The function of these enzymes is largely unknown. Click here for file

Additional file 16 **α-fucosidase I and II Possible Groups of Orthologs (PoGOs) in green plants**. Classification of α-fucosidase I and II PoGOs by taxonomic ranking and the complete list of gene IDs. Click here for file

Additional file 17 **Detailed phylogenetic analysis of α-fucosyltransferases in green plants**. *Arabidopsis Mur2 *PoGO is marked (Figure 5C). The topology was inferred by NJ method with 1000 bootstraps replicates and the genetic distances were calculated using p-distance. Bootstrap values higher than 50% are shown. *Mur2 *are present among spermatophytes and share similarity with uncharacterized gene from *Physcomitrella *and *Selaginella*, suggesting that the genes that gave rise to *Mur2 *orthologs emerged in early land plants. Click here for file

Additional file 18 **α-fucosyltransferase Possible Groups of Orthologs (PoGOs) in green plants**. Classification of α-fucosyltransferase PoGOs by taxonomic ranking and the complete list of gene IDs. Click here for file

Additional file 19 **Detailed phylogenetic analysis of β-galactosyltransferases in green plants**. *Arabidopsis Mur3 *PoGO is marked (Figure 5D). The topology was inferred by NJ method with 1000 bootstraps replicates and the genetic distances were calculated using p-distance. Bootstrap values higher than 50% are shown. Several genes from chlorophytes (27 from *Chlamydomonas*, 16 from *Volvox*, three from *Ostreococcus tauri*, and two from *O. lucimarinus*) could represent the ancestral plant exostosin-like genes from which the XyG galactosyl tranferase activity probably evolved. This analysis includes animal exostosin. Click here for file

Additional file 20 **β-galactosyltransferase Possible Groups of Orthologs (PoGOs) in green plants**. Classification of β-galactosyltransferase PoGOs by taxonomic ranking and the complete list of gene IDs. Click here for file
