# Supplementary figures and images for: Evolution of xyloglucan-related genes in green plants
Source: BMC Evol Biol. 2010 Nov 5;10:341. doi: 10.1186/1471-2148-10-341 (PMC3087550; doi:10.1186/1471-2148-10-341)

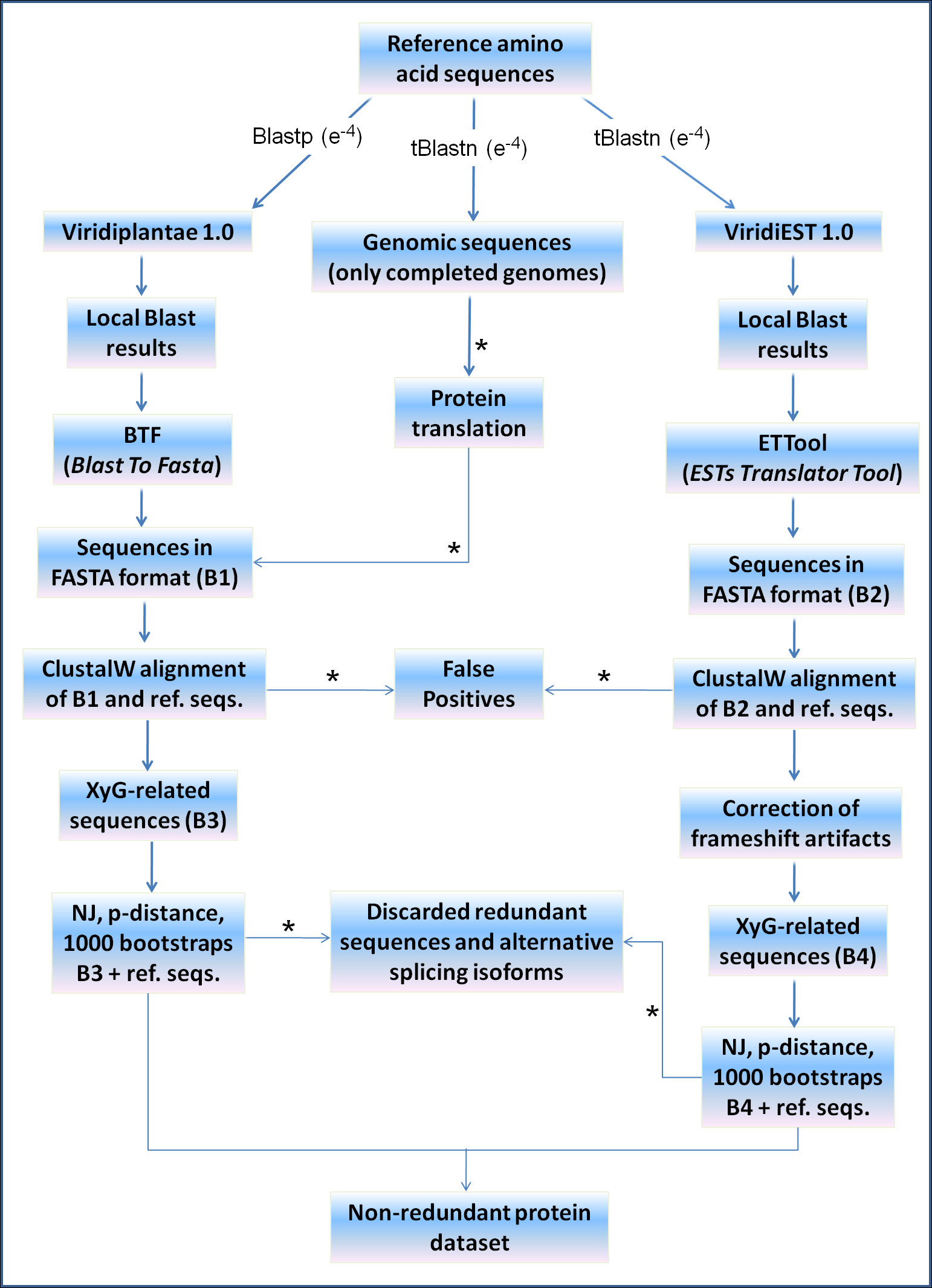

Supplement: Additional file 1 — Bioinformatics search protocol. The pipeline was used in construction of non-redundant protein data-sets. The arrows with asterisks represent manually conducted processes. The e-value cutoffs were 1e-4 for Blastp and tBlastn. Our own programs (BTF and ETTool, both written in JAVA®) were developed for this protocol (available upon request). False positives from B1 and B2 protein sets were eliminated from the alignment by visual confrontation with reference sequences. A NJ tree was generated using B3 and B4 sets together with reference sequences. Redundant sequences and alternative splicing isoforms were eliminated by manual inspection of resulting tree. The final non-redundant protein data-sets obtained were used in our analyses. [file 1471-2148-10-341-S1.TIFF]

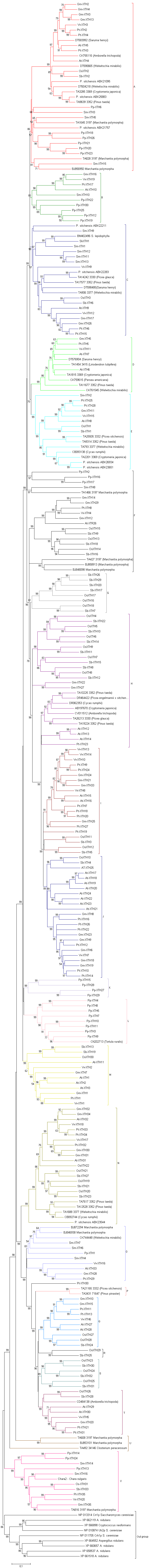

Supplement: Additional file 2 — Detailed phylogenetic analysis of XTH gene family in green plants. PoGOs names and color scheme are the same of Figure 2. The topology was inferred by Neighbor-Joining (NJ) method with 1000 bootstraps replicates and the genetic distances were calculated using p-distance. Bootstrap values higher than 50% are shown. [file 1471-2148-10-341-S2.TIFF]

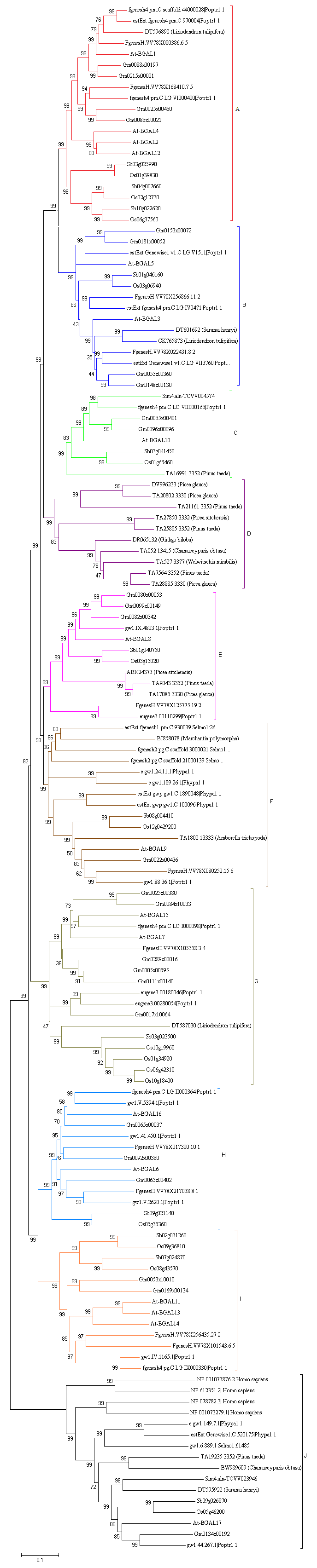

Supplement: Additional file 4 — Detailed phylogenetic analysis of β-galactosidase gene family in green plants. PoGOs names and color scheme are the same of Figure 3A. The topology was inferred by NJ method with 1000 bootstraps replicates and the genetic distances were calculated using p-distance. Bootstrap values higher than 50% are shown. [file 1471-2148-10-341-S4.TIFF]

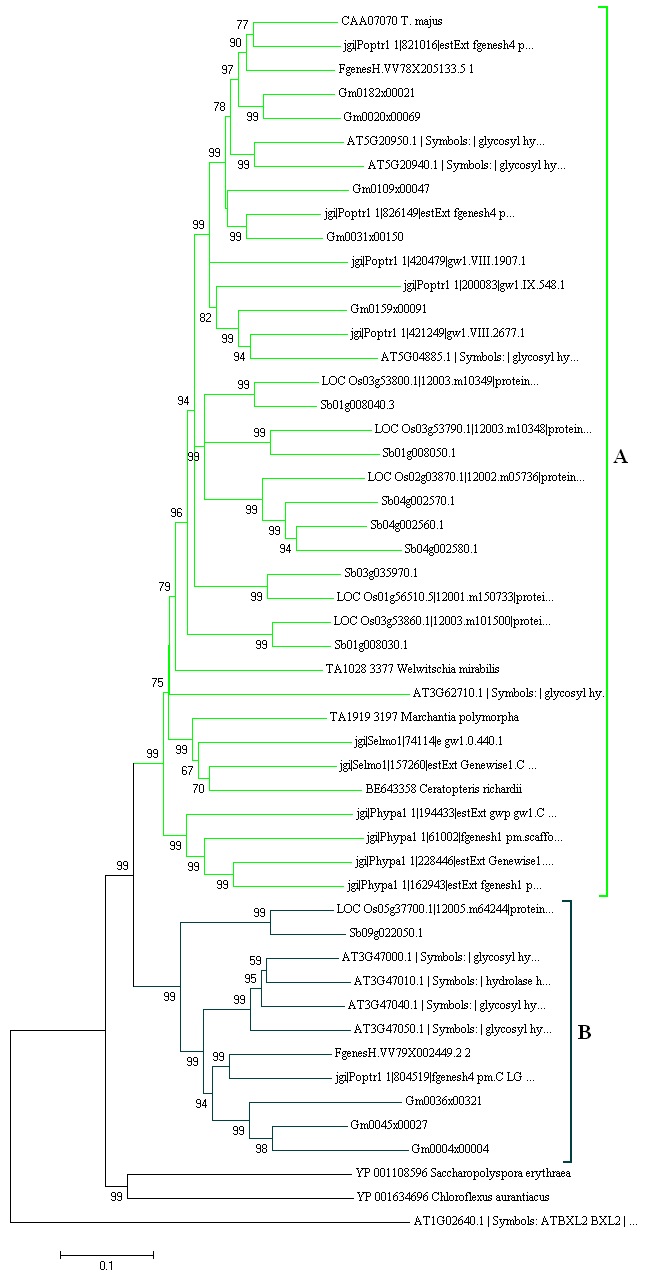

Supplement: Additional file 6 — Detailed phylogenetic analysis of β-glucosidase gene family in green plants. Description: PoGOs names and color scheme are the same of Figure 3B. The topology was inferred by NJ method with 1000 bootstraps replicates and the genetic distances were calculated using p-distance. Bootstrap values higher than 50% are shown. [file 1471-2148-10-341-S6.TIFF]

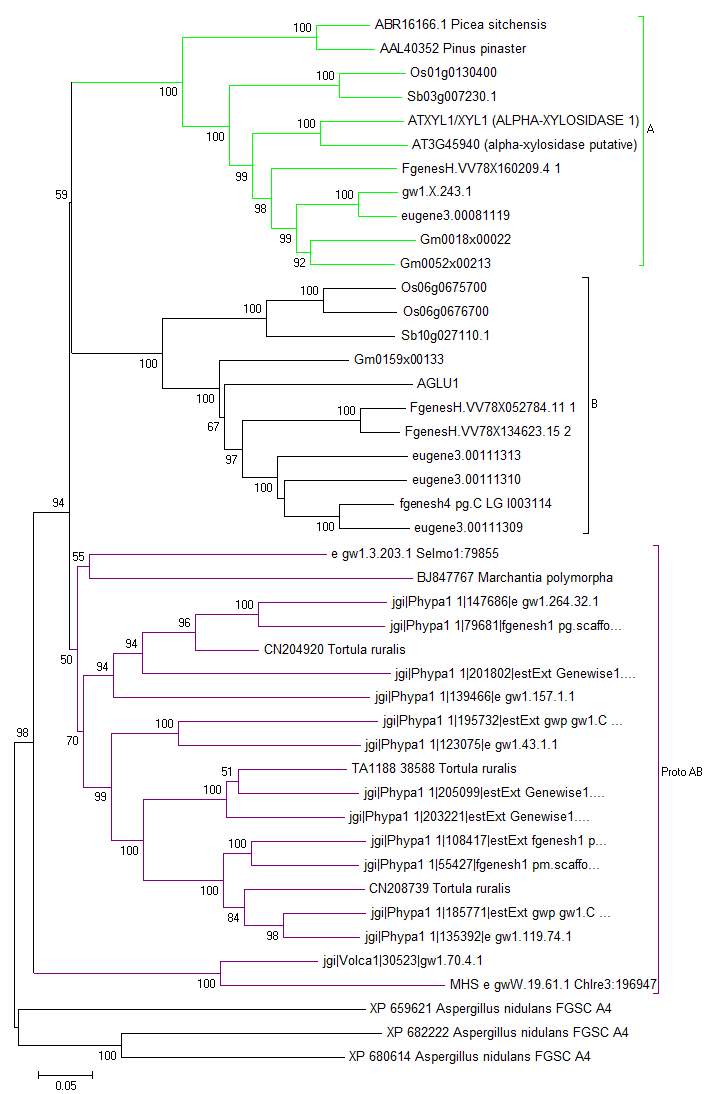

Supplement: Additional file 8 — Detailed phylogenetic analysis of α-xylosidase gene family in green plants. PoGOs names and color scheme are the same of Figure 3C. The topology was inferred by NJ method with 1000 bootstraps replicates and the genetic distances were calculated using p-distance. Bootstrap values higher than 50% are shown. [file 1471-2148-10-341-S8.TIFF]

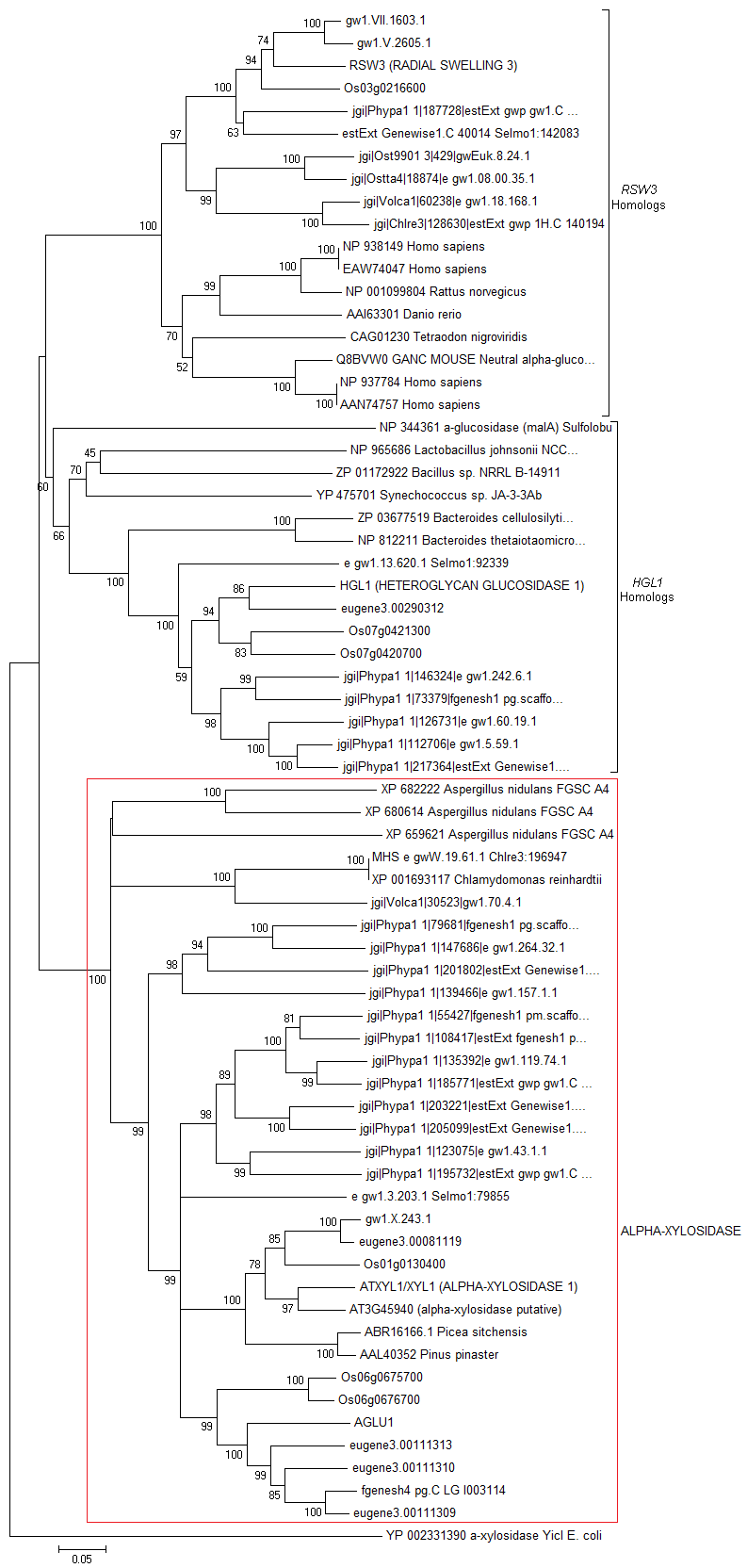

Supplement: Additional file 10 — Phylogenetic analysis of α-xylosidase related homologous groups in Eukaryotes and Bacteria. All sequences analyzed were selected using AtXYL1 from Arabidopsis (At1g68560) as query in blast searches with e-value cutoff of e-4. The topology was inferred by NJ method with 1000 bootstraps replicates and the genetic distances were calculated using p-distance. Bootstrap values higher than 50% are shown. [file 1471-2148-10-341-S10.TIFF]

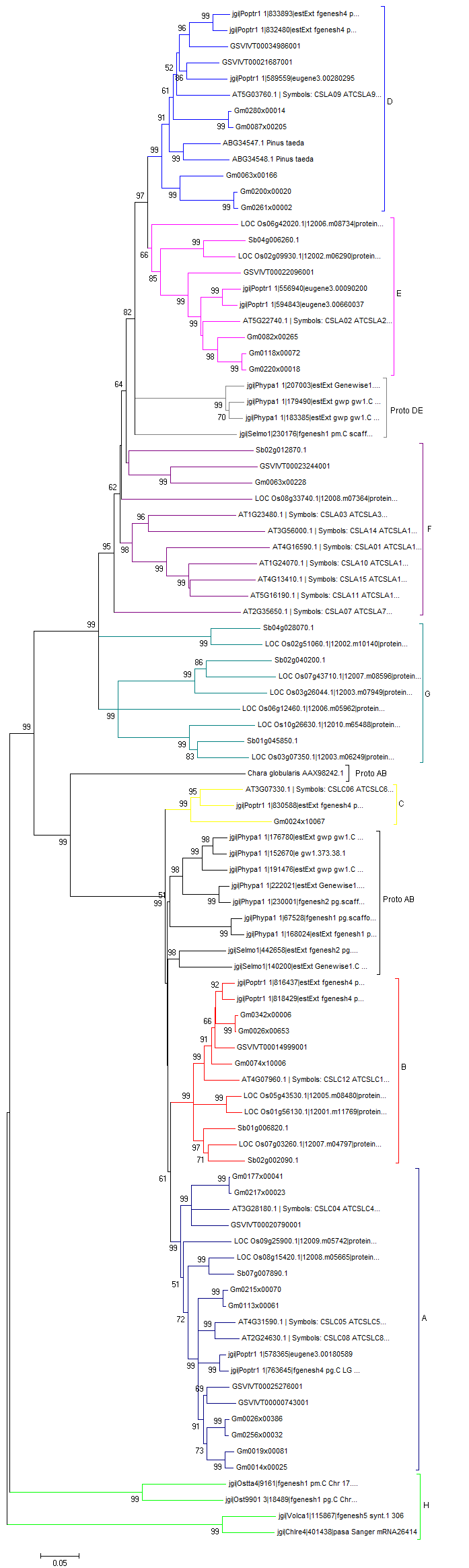

Supplement: Additional file 11 — Detailed phylogenetic analyses of CSL-A and CSL-C (β-Glucan Synthase) gene families in green plants. PoGOs names and color scheme are the same of Figure 4A. The topology was inferred by NJ method with 1000 bootstraps replicates and the genetic distances were calculated using p-distance. Bootstrap values higher than 50% are shown. [file 1471-2148-10-341-S11.TIFF]

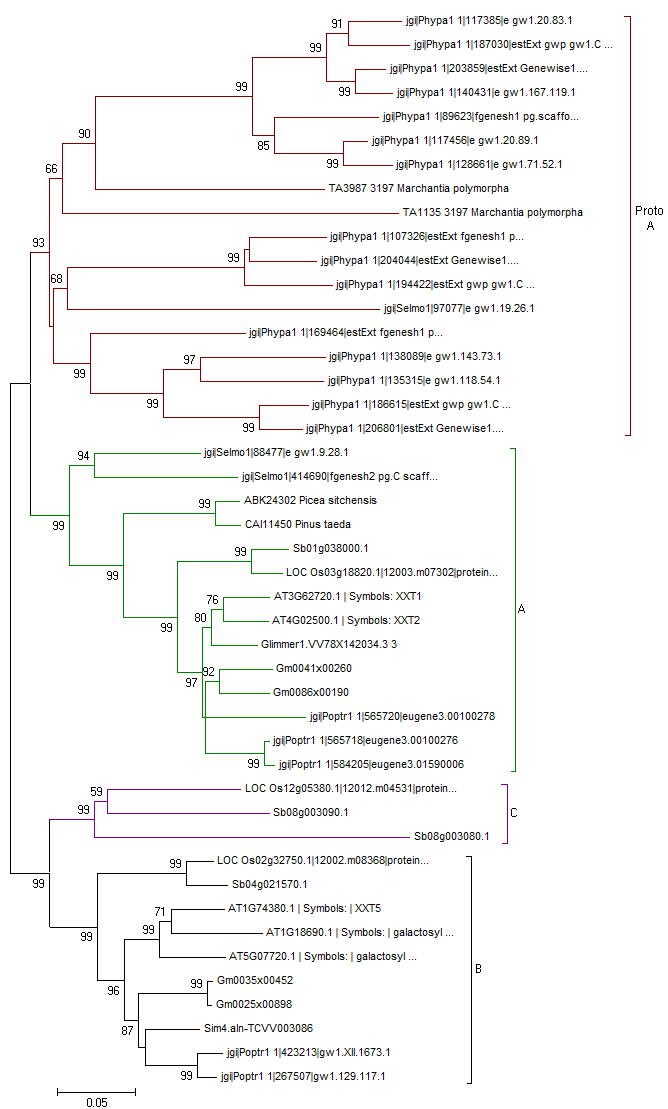

Supplement: Additional file 13 — Detailed phylogenetic analysis of α-xylosyl transferase (XXT) gene family in green plants. PoGOs names and color scheme are the same of Figure 4B. The topology was inferred by NJ method with 1000 bootstraps replicates and the genetic distances were calculated using p-distance. Bootstrap values higher than 50% are shown. [file 1471-2148-10-341-S13.TIFF]

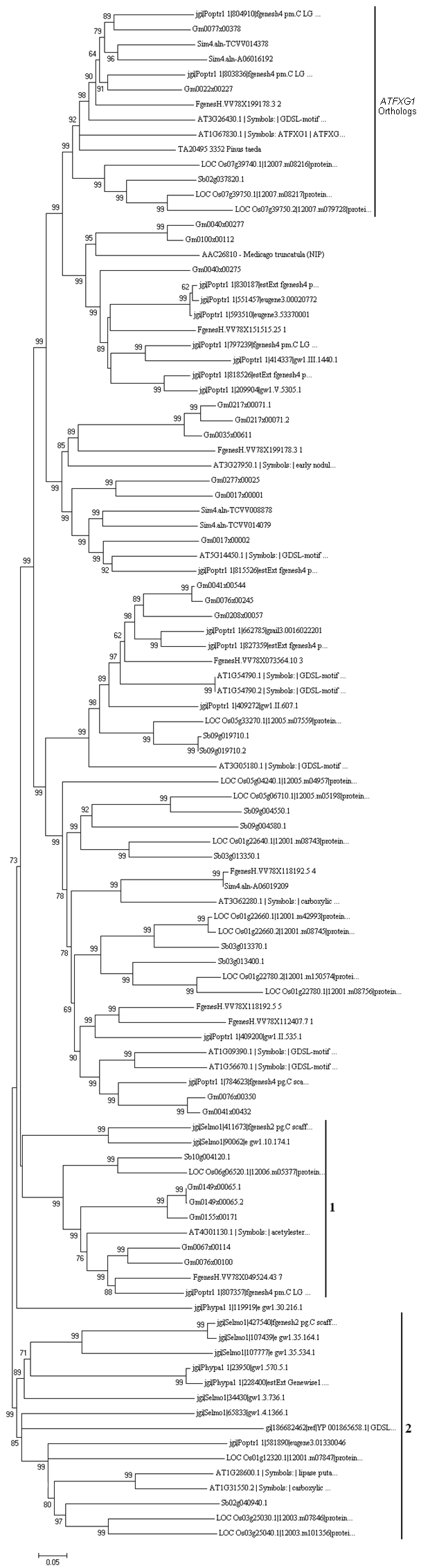

Supplement: Additional file 15 — Detailed phylogenetic analysis of α-fucosidase type I gene family in green plants. AtFXG1 (At1g67830) PoGO is marked (Figure 5A). The topology was inferred by NJ method with 1000 bootstraps replicates and the genetic distances were calculated using p-distance. Bootstrap values higher than 50% are shown. This analysis allowed identification of PoGOs 1 and 2 which integrate Selaginella and Physcomitrella genes, suggesting that they emerged at least in the last common ancestor of land plants and represent the ancestral groups. The function of these enzymes is largely unknown. [file 1471-2148-10-341-S15.TIFF]

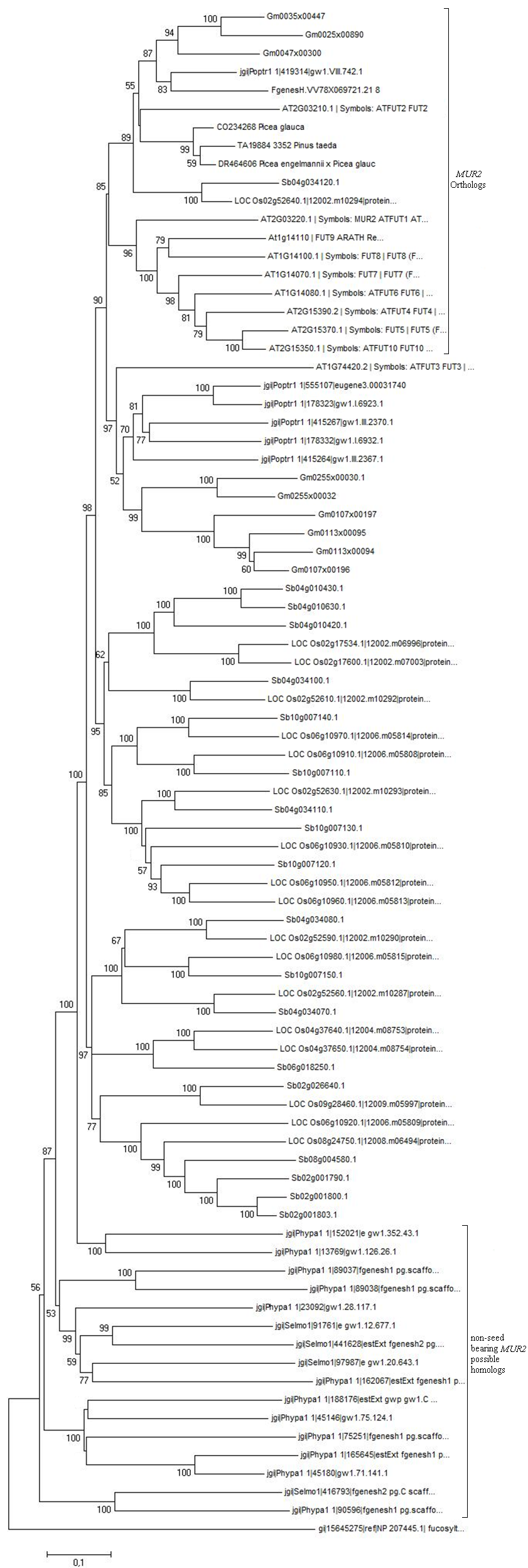

Supplement: Additional file 17 — Detailed phylogenetic analysis of α-fucosyltransferases in green plants. Arabidopsis Mur2 PoGO is marked (Figure 5C). The topology was inferred by NJ method with 1000 bootstraps replicates and the genetic distances were calculated using p-distance. Bootstrap values higher than 50% are shown. Mur2 are present among spermatophytes and share similarity with uncharacterized gene from Physcomitrella and Selaginella, suggesting that the genes that gave rise to Mur2 orthologs emerged in early land plants. [file 1471-2148-10-341-S17.TIFF]

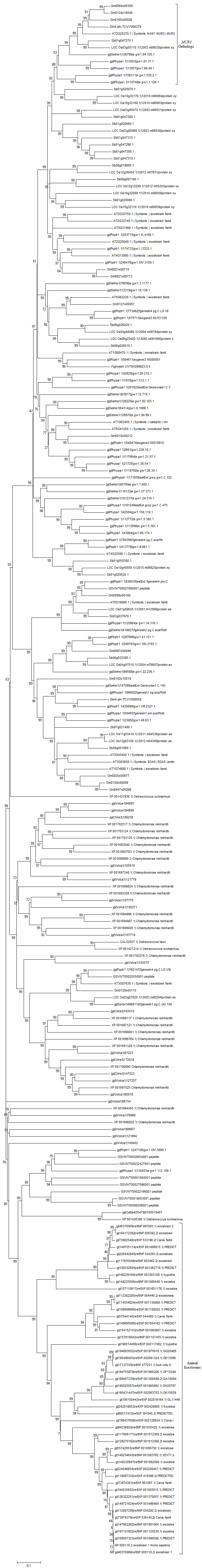

Supplement: Additional file 19 — Detailed phylogenetic analysis of β-galactosyltransferases in green plants. Arabidopsis Mur3 PoGO is marked (Figure 5D). The topology was inferred by NJ method with 1000 bootstraps replicates and the genetic distances were calculated using p-distance. Bootstrap values higher than 50% are shown. Several genes from chlorophytes (27 from Chlamydomonas, 16 from Volvox, three from Ostreococcus tauri, and two from O. lucimarinus) could represent the ancestral plant exostosin-like genes from which the XyG galactosyl tranferase activity probably evolved. This analysis includes animal exostosin. [file 1471-2148-10-341-S19.TIFF]
